# Time to Develop Guidelines for Spiritual Care in Serious Illness

**DOI:** 10.1089/pmr.2024.0035

**Published:** 2024-09-06

**Authors:** Shelley E. Varner-Perez, Amber R. Comer, George Fitchett

**Affiliations:** ^1^ School of Health & Human Sciences, Indiana University, Indianapolis, Indiana, USA.; ^2^ Department of Spiritual Care and Chaplaincy, Indiana University Health, Indianapolis, Indiana, USA.; ^3^ Daniel F. Evans Center for Spiritual and Religious Values in Healthcare, Indiana University Health, Indianapolis, Indiana, USA.; ^4^ Regenstrief Institute, Inc., Indiana University Center for Aging Research, Indianapolis, Indiana, USA.; ^5^ Indiana University School of Medicine, Indianapolis, Indiana, USA.; ^6^ Department of Religion, Health and Human Values, Rush University Medical Center, Chicago, Illinois, USA.

**Keywords:** spirituality, spiritual care, serious illness, chaplain

## Abstract

In 2022, a JAMA systematic review of 342 high quality studies called for spiritual care to be a routine part of care for patients with serious illness. The review’s multidisciplinary panel made several recommendations for addressing patients’ and families’ spiritual concerns. Despite these evidence-based recommendations, there are no clinical guidelines that inform when and how such spiritual care should be provided. We propose convening a multi-disciplinary workgroup to generate specific and actionable guidelines for incorporating spiritual care in serious illness care. We suggest three workgroup priorities: (1) determining best approaches to identifying patient and family members’ spiritual care needs; (2) developing ways to integrate chaplains into routine clinical care; and (3) determining best approaches to communicate availability of spiritual care. Developing these guidelines is an imperative next step to deliver high quality, person and family-centered care.

A 2022 systematic review published in *JAMA* included an examination of research from around the world about spirituality and spiritual care in patients with serious illness.^1^ This review of 342 high quality studies reported strong evidence that spirituality is important for most patients and spiritual care is frequently desired by them. It found that spiritual concerns were common among these patients and frequently unaddressed. Addressing spiritual concerns during health care delivery contributed to the provision of higher quality health care, including clinically meaningful reductions in anxiety, improved spiritual well-being, and better end-of-life outcomes.^1–2^ Conversely, the review reported, unaddressed spiritual concerns were associated with poorer outcomes including quality of life. This is particularly relevant for Black and Hispanic patients in the United States who, compared to White patients, rate religion and spirituality as more important in coping with serious illness.^1,3^ Utilizing a biopsychosocial spiritual approach incorporates multiple domains and elicits patients’ and families’ values and preferences for medical treatment.^4^ Although the evidence supports incorporating spiritual care into critical illness care, there are no guidelines or standardized approach for addressing spiritual concerns during serious illness care, leaving a formidable gap in an important care domain.

## What Is Spirituality and How Is It Addressed During Patient Care?

Spiritual concerns arise from spirituality, which is broader than religion. Spirituality includes a search for meaning, including but not limited to connection with the transcendent, other living beings (human or animal), and the natural world.^5^ Spirituality may inform a person’s worldview and daily practices (such as meditation or prayer) and contribute to a sense of purpose or mindfulness. At times, spirituality extends to existential considerations.^6^

Chaplains are board-certified health care professionals with Master-level education and specialized training to address spiritual concerns in clinical contexts. As members of the health care team and employees of health care systems, chaplains navigate the complexity and nuance of diverse spiritualities and their impact on patient’s medical decisions, family dynamics, and the care plan. Although chaplains are colloquially thought of as being oriented toward a certain religion, this is a misunderstanding. Chaplains are trained to serve patients and families of all worldviews, including patients and families who are nonreligious, humanist, agnostic, or unaffiliated. Chaplains follow professional ethical guidelines, including nonproselytization.

Current practice guidelines for family-centered care in the Intensive Care Unit (ICU), developed by the American College of Critical Care Medicine, encourage offering chaplain services to ICU patients and their families.^7^ These guidelines do not specify the timing or frequency of spiritual care provision, and they limit spiritual care to those with an “expressed desire.” In practice, spiritual care is often only offered near end-of-life, missing opportunities to engage patients and families earlier in their hospitalization and disease course.^8^ Earlier introduction of spiritual care has been shown to mitigate quality of life concerns and proactively address spiritual needs, benefiting patients, families, and the health care team when navigating potential differences around topics such as hope for a miracle.^2,9,10^ Because chaplains have clinical training, utilizing a chaplain instead of or in tandem with community clergy for this type of spiritual advising is crucial for addressing the spiritual care gap in clinical care.

## Gaps in Current Clinical Practice Guidelines

The *JAMA* review mentioned above identified three major gaps in current clinical practice related to spiritual care: (1) spiritual care is not routinely incorporated into the care for patients with serious illness; (2) spiritual care education is not incorporated into the training of interdisciplinary teams caring for persons with serious illness; and (3) specialty practitioners of spiritual care, such as chaplains, are not being routinely incorporated into the care of patients with serious illness.^1^ The expert panel reviewing the study’s results advocated for incorporating spiritual care as a *routine* (emphasis added) aspect of serious illness care. Although a growing body of evidence supports incorporating spirituality during clinical care, clinical practice guidelines do not provide guidance on when and how spirituality should be addressed. This is a disservice to patients and families who want to have their spiritual concerns addressed^1^ and likewise to providers who want to provide appropriate care. Identifying specific recommendations for addressing spirituality in routine clinical practice is a crucial next step.

## Recommendations

There are no clinical practice guidelines for addressing spirituality in seriously ill adult patients. As the *JAMA* review reported, unaddressed spiritual concerns can lead to unresolved spiritual and physical pain, unnecessary distress, and preventable suffering.^1^ Considering the evidence that supports incorporating spiritual care into serious illness care, it is crucial to convene a workgroup that will generate specific and actionable guidelines for incorporating spiritual care in serious illness care. We suggest three workgroup priorities, which were derived from the *JAMA* systematic review: (1) determine best approaches to identifying patient and family members spiritual care needs; (2) develop ways to integrate chaplains into routine clinical care to address the spiritual needs of patients; and (3) determine best approaches to communicate with families about the availability of spiritual care.

The workgroup should include representatives from key disciplines, including medicine, chaplaincy, and nursing. International representatives should be included as the challenges for providing spiritual care are similar in many nations. It should include members from diverse racial, ethnic and religious/spiritual backgrounds. The work group should follow the principles of the CMSS^11^ or similar guidelines. The resulting guidelines should be widely adopted and implemented in serious illness clinical settings.

## Conclusion

The recent *JAMA* systematic review makes important recommendations for addressing spirituality in serious illness. Additional work is needed to turn these recommendations into guidelines for addressing spiritual concerns for patients experiencing serious illness and their families. These next steps are imperative for delivering high-quality, person- and family-centered care. We advocate forming a workgroup to create clinical guidelines to address these crucial gaps, and we identify three evidence-based priorities for the workgroup to tackle. It is time to guide our teams and organizations to address spiritual concerns that matter to patients and families and to do so effectively, efficiently, and professionally following evidence-based guidelines.

## Abbreviations Used

CMSS: Council of Medical Specialty Societies
JAMA: Journal of the American Medical Association
